# Perceived Risk of COVID-19 and Employee Decision-Making: How Psychological Distress during the Pandemic Increases Negative Performance Outcomes among Healthcare Workers

**DOI:** 10.3390/ijerph19116762

**Published:** 2022-06-01

**Authors:** Ngqabutho Moyo, Anita D. Bhappu, Moment Bhebhe, Farai Ncube

**Affiliations:** 1 Department of Management of Complex Systems, School of Engineering, University of California, 5200 North Lake Rd., Merced, CA 95343, USA; abhappu@ucmerced.edu; 2 Department of Human Resource Management, Faculty of Social Sciences, Midlands State University, Gweru P. Bag 9055, Zimbabwe; mbhebhe@staff.msu.ac.zw (M.B.); fncube@staff.msu.ac.zw (F.N.)

**Keywords:** frontline workers, employee disengagement, low morale, turnover intention, burnout, survey research, healthcare employees

## Abstract

In this research, we examined how COVID-19 impacts employee decision-making and performance, knowing that this virus has negatively affected public health, crippled economies, and transformed social and business environments across the globe. To quantitatively test our specific hypotheses regarding the effects of employees’ perceived risk of COVID-19 and psychological distress on negative performance outcomes, we surveyed 443 healthcare workers who were employed by a group of private hospitals in Zimbabwe. These essential workers were delivering day-to-day frontline services with high exposure to COVID-19 during the pandemic. We find that employees’ perceived risk of COVID-19 increases their disengagement, turnover intention, burnout, and low morale at a *p* < 0.05 significance level. These latter relationships are mediated by employees’ psychological distress at a *p* < 0.05 significance level. Our findings shed light on how the COVID-19 pandemic is affecting the cognitions and behaviors of the frontline workers who are vulnerable to this contagious disease. Turnover intentions are amplified among healthcare employees, due to their perceived risk of COVID-19 and the resulting psychological distress. Similarly, burnout becomes predominant as these workers worry about contracting the coronavirus due to the poor working conditions they face. As such, our research confirms that the pandemic has intensified the precariousness of work and challenge of managing employee performance, especially for frontline healthcare workers.

## 1. Introduction

The novel coronavirus, also known as COVID-19, is a highly transmittable and pathogenic disease [1]. It has instigated fear and caused panic all over the globe [2,3,4,5,6,7,8,9,10,11,12,13,14,15], negatively affected public health, crippled economies, and transformed social and business environments [16]. The COVID-19 pandemic, which is regarded as one of the worst pandemics in human history [17], has also had an unprecedented effect on the living conditions and deaths of human beings. Globally, as of February 2022, more than 400 million cases of COVID-19 had been confirmed, including almost 6 million deaths, even though more than 10 billion doses of vaccines had been administered [18]. Control measures, such as mandatory lockdowns and social distancing, have also affected the mental health of the public at large [19].

In response to the COVID-19 pandemic, most employers have been implementing measures such as the washing of hands, use of hand sanitizers, closure of operations, and social distancing [20,21]. Many have also adopted hybrid and remote work arrangements [22,23,24], which allow employees to be geographically dispersed and physically distanced [25]. While hybrid and remote work appears to have become a new normal in many corporations [26], employees in essential sectors, such as healthcare, have been required to increase their work hours and physical presence, in order to support operational demands. Most healthcare employees, particularly nurses, not surprisingly, are more psychologically disturbed and overworked. Some employees have also been exhibiting negative performance outcomes, such as burnout, disengagement, psychological distress, and low morale [27].

**Figure 1:**
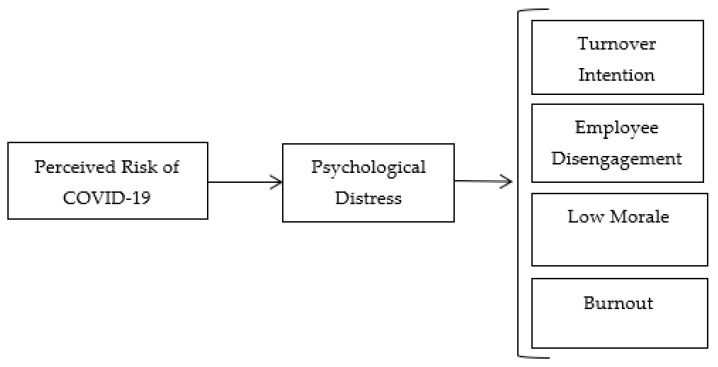
Theoretical model.

Globally, employee engagement decreased by 2%, from 22% in 2019 to 20% in 2020 [28], amid the COVID-19 pandemic. Around 45% of employees indicated that their lives had been significantly affected by the coronavirus, and workers’ daily stress levels reached a record high, increasing from 38% in 2019 to 43% in 2020 [28]. Therefore, understanding how these factors affect employee decision-making should be a priority for organizations because their effectiveness is highly dependent on employees’ willingness to perform [29,30]. Furthermore, organizations should be looking for better ways to adapt to complexity in their environment [31]. Amid the COVID-19 pandemic and all other infectious disease outbreaks, employers ought to protect their employees and cater to their wellbeing because they are the most valuable assets of an organization. When employees are treated well, they become loyal, engaged, committed, and attached to their organization [32]. Therefore, this research examines how COVID-19 impacts employee decision-making processes and performance outcomes. Specifically, we investigate how employees’ perceived risk of COVID-19 influences their psychological distress, disengagement, turnover intention, burnout, and low morale. Figure 1 depicts our theoretical model, which we now situate within the broader literature on employee behavior.

### 1.1. COVID-19 Pandemic and Employee Behavior

The COVID-19 pandemic has disrupted normative work arrangements and influenced employee behavior in many ways [33]. It has been associated with job insecurity, financial losses, social exclusion, and stigmatization [20], as well as uncertainty about the future of work, lower job attitudes, and performance [34,35]. Globally, it remains the major workplace transformative event that has forced many organizations to adopt new work arrangements, such as allowing employees to work from home [33]. However, employees in essential sectors, such as healthcare, had to increase their working hours and physical presence, in order to meet operational demands [36]. Healthcare employees have complained about the scarcity of resources, insufficient support, and poor leadership and communication during the COVID-19 pandemic [37].

The COVID-19 pandemic has caused severe psychological effects among healthcare workers [38,39,40,41]. Although it resulted in the employment of more medical practitioners and increased medical research funding [21], healthcare workers are more vulnerable, since they work in an environment where COVID-19 infections are more likely to occur [42,43]. As a result, healthcare workers have experienced emotional fatigue, aggression, and depersonalization [44,45]. In fact, most employees have been exhibiting mood swings, depressive thoughts, headache and gastric disorders, isolation, demotivation, and poor performance at work [46]. In addition, employees have been experiencing psychological distress, which has been associated with exhaustion and cynical attitudes [15,47,48]. Prolonged exposure to stress and inadequate coping strategies can result in emotional exhaustion [49].

### 1.2. Employee Perceived Risk & Psychological Distress

The perceived risk of COVID-19 is regarded as a key driver of psychological distress [20] because it is associated with a wide range of stressors that drain the mental health of employees, especially fear and panic [20]. Other stressors include the threat of infection [50], uncertainty [51], quarantine and confinement [52], exclusion from the society and stigmatization [52], job insecurity, and loss of finances [53]. Employees in the health sector have been the most affected frontline workers during the pandemic [54]. This has been largely attributed to their level of exposure, given the nature of their job [55] and risk of interacting with patients suffering from COVID-19 [56], as well as the risk of being infected by work colleagues [57]. It is, therefore, important to understand how the perceived risk of COVID-19 influences employee decision-making.

Perceptions regarding the risk of a disease, also known as the perceived susceptibility and severity, affect an individual’s behavior [58]. Deciding on whether or not to adopt safe precautions is highly dependent on people’s perceptions of their vulnerability to illnesses [58]. According to the Health Belief Model (HBM), one of the well-established models of health behavior, perceived risk or severity can be understood as a person’s subjective assessment of the seriousness of a disease, which is affected by different types of factors, such as future expectations and current reality [59]. An increased perception regarding disease severity is associated with proactive precautious health behaviors [58,60]. Individuals who trust that they are not at risk of falling ill are less likely to take safe precautions, thereby exposing themselves and others to hazard, compared to those who strongly believe that they are at risk [61].

The perceived risk of COVID-19 has challenged the psychological resilience of workers [62] and increased their psychological distress [20,38,63]. Based on Lazarus and Folkman’s (1984) transactional stress model, threatening situations, such as pandemics, ignite anxiety [63]. On a similar note, an emphasis of the influential role played by a situation in building anxiety is made by Cheng and McCarthy’s (2018) theory of workplace anxiety, as well as Gross’ (1998) process framework of emotion regulation [63,64,65]. Psychological distress is defined as the state of person’s emotional suffering, consisting of symptoms of depression, such as sadness and anxiety [66], as well as somatic symptoms, such as insomnia [20,66]. It is an indicator of mental health problem [66] because it may result in major depression if not identified [20,66]. Psychological distress is triggered by a person’s inability to cope with a situation outside of their control [66], such as the COVID-19 pandemic. As such, we propose that:

**Hypothesis** **1.** *Employees’ perceived risk of COVID-19 increases their psychological distress.*

### 1.3. Employee Perceived Risk & Negative Performance Outcomes

The conservation of resources (COR) theory suggests that stress arises when (a) there is a threat of losing essential resources, (b) there is loss of key resources, and (c) an effort to achieve central or key resources has been made, but no resources have been attained [67]. In this regard, a sense of purpose and meaning in life, family, health, wellbeing, self-esteem [67], and social support are among the frequently valued resources [68]. When these resources are exhausted, employees tend to enter a defensive mode, in order to preserve themselves and guard against aggressive, duplicitous, and irrational behavior [67]. When threatened, individuals tend to use a coping strategy, in order to overcome the threat [63]. For instance, when feeling anxious, they are likely to develop a defense mechanism, in the form of a fight or flight response, as a way of overcoming the threat [69]. A fight response is activated when a threat is deemed manageable, whereas a flight response is ignited when a threat is hard to overcome [70].

COVID-19 is a highly contagious disease, which can cause severe health problems, such as abdominal pain, pneumonia [71,72], and even death [73]. Most workers are worried that they might get infected, be stigmatized at work, infect their relatives and coworkers, and lose personal freedoms [27,74,75] because the virus has no cure [76,77]. Therefore, the perceived risk of COVID-19 is believed to induce a flight response [63] and negative performance behaviors among workers [78]. Organizations tend to experience increased levels of absenteeism and poor work performance during epidemics and pandemics [79]. Stress and poor working conditions during the COVID-19 pandemic have increased negative performance outcomes [80,81,82], such as employee turnover intention, disengagement, low morale, and burnout [24,83,84,85].

Turnover intention is defined as the probability that a worker will quit an organization [86,87]. When employees quit their jobs, organizations tend to bear the loss of human capital. They also suffer from the costs associated with the loss of productivity [88,89]. Turnover intention is usually attributed to work-related factors, such as poor working conditions, the lack of safety at work, and individual and external factors [90]. Amid the COVID-19 pandemic, turnover intention among employees has been influenced by higher levels of psychological distress [37,91]. Psychological distress is the key driver of increased turnover intentions at work [83,92,93]. In addition, the perceived risk of COVID-19 has been examined, in relation to employees’ wellbeing and mental health outcomes [94]. The perception of this disease as a serious threat increases the fear of COVID-19 among healthcare workers, hence their intention to leave [95,96]. As such, the pandemic is forcing workers to think about quitting their jobs.

Due to the pandemic, most workers have been exhibiting a lack of engagement [83]. Disengagement is concerned with the lack of motivation and attachment towards the achievement of organizational goals and objectives [97,98]. The risk and fear of contracting COVID-19 has also resulted in low morale among employees [99]. Employee morale is regarded as the epitome for business success and a key antecedent of achieving organizational competitiveness. Low morale among employees is viewed as a threat by organizations that seek to achieve their goals and objectives [100].

Amid the COVID-19 pandemic, most employees have also been experiencing high levels of burnout [1,101]. Burnout is defined as a syndrome that emanates from the chronic stress at work, with adverse effects on employees’ psychological well-being [102], as well as their work behavior and physical health [103]. Burnout is usually attributed to hostile working conditions, which result from prolonged stress at work, with negative effects on employee performance [104]. As a result of burnout, employees may display behaviors such as negative attitudes, lack of commitment, dissatisfaction, and poor performance at work [102]. As such, we propose that:

**Hypothesis** **2.** *Employees’ perceived risk of COVID-19 increases their negative performance outcomes.*

**Hypothesis** **3a.** *Psychological distress mediates the relationship between employees’ perceived risk of COVID-19 and turnover intention.*

**Hypothesis** **3b.** *Psychological distress mediates the relationship between employees’ perceived risk of COVID-19 and disengagement.*

**Hypothesis** **3c.** *Psychological distress mediates the relationship between employees’ perceived risk of COVID-19 and low morale.*

**Hypothesis** **3d.** *Psychological distress mediates the relationship between employees’ perceived risk of COVID-19 and burnout.*

## 2. Materials and Methods

### 2.1. Research Methods

We conducted an employee survey, with a high response rate, to test our theoretical model and hypotheses, which is an appropriate method [105]. Quantitative research allows researchers to clearly and precisely specify both the independent and dependent variables under investigation. We first used SPSS Version 20 software to enter our survey data, analyze sample characteristics, and measure correlations. We then used Smart PLS3 software to construct and evaluate our measurement model using structural equation modeling, which is an appropriate statistical method [106]. In our quantitative analyses, perceived risk of COVID-19 was the independent variable, whereas turnover intention, employee disengagement, low morale, and burnout were the dependent variables; psychological distress was the mediator.

### 2.2. Survey Sample

We surveyed healthcare workers employed by a group of private hospitals in one of the largest provinces in Zimbabwe. They were essential workers who were actively delivering day-to-day frontline services [107] with high exposure to COVID-19, given the nature of their work, during the pandemic. Our printed survey was made available to healthcare workers in four different hospitals by their employers for voluntary completion. We, therefore, used a convenience, non-probability sampling method [108,109] to select survey respondents. Survey respondents also remained anonymous to us because we collected their completed unidentified surveys from their employer’s central office, where they had dropped them off. We, therefore, had no data to assess differences across the four hospitals, although none were anticipated, given the convergence of management practices within the hospital group. In total, 443 of 550 hospital employees completed our survey, representing an 80.5% response rate. Given anonymity requirements, we did not collect data on these employees’ specific job roles and, therefore, had no data to assess differences across staff types.

### 2.3. Survey Measures

To measure the perceived risk of COVID-19, we adopted a scale (see Table 1) developed by [110]. This scale is regarded as valid and acceptable for measuring employees’ perceived risk of COVID-19 given its reliability score of 0.86 [111]. Likert responses to scale items were: 1 = strongly disagree; 2 = disagree; 3 = neutral; 4 = agree; 5 = strongly agree.

To measure psychological distress, we adapted the Perceived Stress Scale, which has reported reliability scores between 0.65 and 0.86 (see Table 1) [112,113]. Likert responses to scale items were: 1 = strongly disagree; 2 = disagree; 3 = neutral; 4 = agree; 5 = strongly agree.

To measure turnover intention, we adopted a scale (see Table 1) developed by Mobley, Horner, and Hollingsworth [86]. This scale is regarded as valid and acceptable for measuring employees’ turnover intention, given its reliability score of 0.91 [111]. Likert responses to scale items were: 1 = strongly disagree; 2 = disagree; 3 = neutral; 4 = agree; 5 = strongly agree.

To measure employee disengagement, we adapted a scale (see Table 1) developed by [114]. Likert responses to scale items were: 1 = strongly disagree; 2 = disagree; 3 = neutral; 4 = agree; 5 = strongly agree.

To measure low morale, we developed a scale (see Table 1) based on our review of the literature. Likert responses to scale items were: 1 = strongly disagree; 2 = disagree; 3 = neutral; 4 = agree; 5 = strongly agree.

To measure burnout, we adopted Maslach’s burnout inventory scale (see Table 1) for human services [84,115]. This scale is regarded as valid and acceptable for measuring employees’ burnout [111]. Likert responses to scale items were: 1 = strongly disagree; 2 = disagree; 3 = neutral; 4 = agree; 5 = strongly agree.

## 3. Results

### 3.1. Sample Characteristics

Out of the 443 survey respondents, 220 (49.7%) were males, and 223 (50.3%) were females. In terms of age, 288 (65%) were between 21 and 30 years old, 152 (34.3%) were between 31 and 40 years old, and 3 (0.7%) were 41 to 50 years old. With regards to education, 310 (70%) had a high school diploma, and 133 (30%) had a bachelor’s degree. In terms of employment history, 151 (34.1%) had less than six years of service with their current employer, whereas 292 (65.9%) had more than six years of service.

### 3.2. Measurement Model Assessment

We initially calculated sample descriptive statistics and Pearson correlations for the variables measured in our survey, which were all significantly correlated, as shown in Table 2.

**Figure 2:**
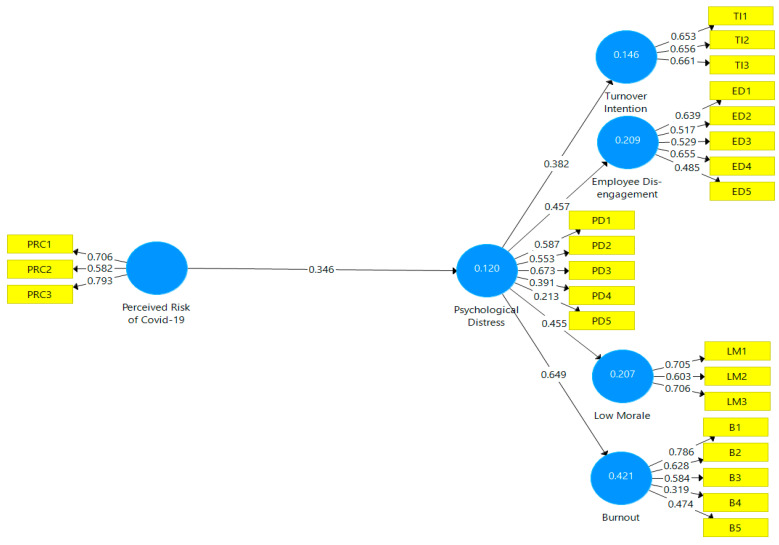
Assessment of measurement model.

Next, we conducted an assessment of our measurement model, in line with the endorsements of well-known researchers, in partial least squares analysis [116]. We first assessed the composite reliability and discriminant validity of each survey scale using the PLS algorithm. The outer loading of each scale item, which should range between 0.40 and 0.70 [105,106], were first examined to determine whether they were measuring the same construct. Out of 27 scale items, measuring 6 variables in our model, 3 of the items from the perceived risk of COVID-19 scale were deleted, as a result of poor factor loadings; these were items 4, 5, and 6 in Table 1. As such, our final calculations were based on the remaining 24 scale items, with most of the factor loadings exceeding the recommended 0.50 threshold [117], as shown in Figure 2.

Composite reliability is the best statistical approach to use when evaluating the internal consistency a scale [105]. Based on the results of the PLS algorithm, the composite reliability of each survey scale had an acceptable level of internal consistency, as recommended by [105]. Specifically, the composite reliability of burnout was 0.701, perceived risk of COVID-19 was 0.739, employee disengagement was 0.703, low morale was 0.712, psychological distress was 0.613, and turnover intention was 0.695.

Discriminant validity evaluates the uniqueness of a measured construct and its representation as a distinct phenomenon of interest in a structural equation model [106]. To assess the discriminant validity, most researchers rely on Heterotrait–Monotrait ratio (HTMT) [118]. Discriminant validity is attained when the obtained values of HTMT are less than the suggested thresholds of 0.85 [119,120] to 0.90 [121]. A value that is higher than this suggested threshold implies that there is lack of discriminant validity [121]. As shown in Table 3, we assessed HTMT for all survey scales by running our first model (PLS algorithm) to calculate these values.

### 3.3. Hypotheses Testing

**Figure 3:**
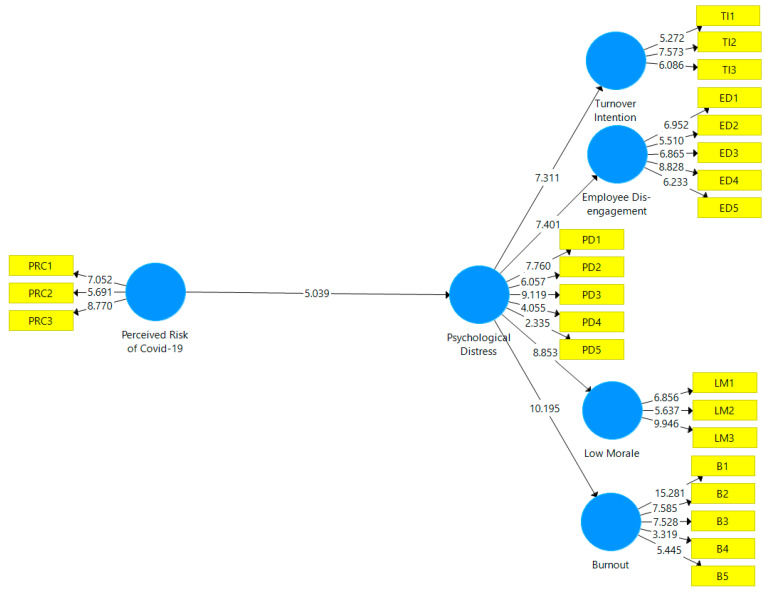
Hypotheses testing.

To test our hypotheses, we then assessed our theoretical model using a regular bootstrapping technique, which was applied to the data from the 443 survey respondents, in order to determine the significance level of the path coefficients shown in Figure 3. Hypotheses 1 and 2 were supported, given the statistically significant direct effects outlined in Table 4. To test Hypotheses 3a–3d, we also assessed the mediation effect of psychological distress. Hypotheses 3a–3d was supported, given the statistically significant indirect effects outlined in Table 5.

## 4. Discussion

The main aim of this research was to examine how COVID-19 impacts employee decision-making processes and performance outcomes. In support of our hypotheses, we find that employees’ perceived risk of COVID-19 increases their disengagement, turnover intention, burnout, and low morale. These latter relationships are mediated by employees’ psychological distress. Our findings shed light on how the global pandemic is affecting the cognitions and behaviors of frontline workers. We find that as healthcare employees’ perception of the risk of COVID-19 increases, their psychological distress surges. This in turn decreases healthcare employees’ work performance and increases their intent to quit their job given working conditions during the pandemic.

Our study establishes that higher perceived risk of COVID-19 and psychological distress increases employee disengagement. During the pandemic, most workers are fearful of contracting this contagious disease that can lead to extreme health problems or death [73,122], which may potentially be fueling disengagement among employees. Previous research indicates that the perceived risk and fear of contracting the virus results in demotivation and low morale among employees [1]. Additionally, workplace absenteeism is usually caused by felt psychological pressure to meet work demands, job insecurity, excessive workloads, and long working hours [123]; these factors were dominant at the time when we conducted this survey research during the pandemic.

The findings of our research also suggest that higher perceived risk of COVID-19 and induced psychological distress are associated with burnout among healthcare employees. Burnout is usually caused by prolonged stress at work and attributed to hostile working conditions, which have adverse effects on employee performance [104]. In other words, there is a connection between burnout and working conditions, which can cause employees to display negative attitudes, lack of commitment, apathy, dissatisfaction, and poor performance at work [102].

Prior research indicates that most workers are experiencing high levels of burnout [1] and suffering from depression and anxiety [124] amid the pandemic. Adjusting to new work schedules, such as rotating shifts, night shifts, and flextime, has also resulted in employee absenteeism and increased turnover intentions [125]. In addition, there has been an increase in alcohol consumption and gambling [126] during the pandemic, especially among the employees forced to work remotely from home [127]. Concern over the risk of contracting the virus is another factor contributing to stress and anxiety among frontline workers [128]. For example, the pandemic has increased a sense of job insecurity among hospitality workers and their perception of being unemployed, thus adversely affecting their mental health [126]. In the construction industry, it has affected employees’ general wellbeing [127]. Even students have experienced boredom, anxiety, and frustration as a result of COVID-19 disruptions in higher education [127]. Thus, the continued mutation of the coronavirus will likely contribute to an increase in the stress levels and resignation of healthcare workers.

### 4.1. Limitations

A limitation of our study is related to our research methodology, which may reduce the generalizability of our findings. We opted for a quantitative approach to conduct this investigation and encountered some issues with our measures. Some of our HTMT values exceeded the suggested threshold of 0.90 [121]. This could be attributed to factors such as mono-method bias, given our cross-sectional data collection using a single survey and response bias, wherein some respondents may have inaccurately or untruthfully completed the survey. Furthermore, our measure of perceived risk of COVID-19 could be expanded to include a cognitive component in addition to the current affect-based questions, similar to the construction worker risk perception (CoWoRP) scale [128]. Finally, we gathered data from only healthcare workers who were more exposed and susceptible to coronavirus than other types of employees.

### 4.2. Future Research

Future research should use a mixed methods approach to incorporate respondents’ qualitative insights on how COVID-19 is influencing their cognitions and behaviors at work, which would provide a more in-depth understanding of employee performance outcomes during the pandemic. Our survey findings should also be replicated with other groups of frontline employees in the retail, agriculture, manufacturing, and mining industries who may be experiencing equal or greater risks during the pandemic than healthcare workers.

### 4.3. Practical Recommendations

Our study reveals that psychological distress mediates the effect of perceived risk of COVID-19 on employees’ turnover intention, disengagement, low morale, and burnout. We, therefore, recommend that organizations help their employees manage pandemic-related stress by providing psycho-social support through counseling and wellness programs. While this may require additional investment, it should limit negative employee performance outcomes and related costs during this uncontrollable pandemic. Furthermore, employees should feel more motivated and engaged when they are reassured that their employer is trying to help them cope in a difficult circumstance.

To improve working conditions and reduce employees’ burnout, we also recommend that organizations provide their workers with additional resources, such as personal protective clothing and products, health insurance, and medical leave, as well as financial compensation for working extended and extra hours. With such resources, employees should be able to pay more attention to and focus on job details, despite their perceived risk of COVID-19, which will enhance their work performance during the pandemic. Organizations should also conduct risk assessments in order to determine if their employees are exposed to any possible threats, and review their safety rules in order to ensure an effective response to infectious diseases outbreaks.

## 5. Conclusions

We conclude by affirming that the COVID-19 pandemic has led to unprecedented effects in the world of work, especially among frontline healthcare employees who are most vulnerable to this contagious disease. Our research findings establish that turnover intentions are amplified among this group of workers due to their perceived risk of COVID-19 and induced psychological distress. The latter reduces employee morale and engagement, with burnout becoming predominant as workers worry about contracting the coronavirus due to the poor working conditions that frontline employees face. As such, our research confirms that the pandemic has intensified the precariousness of work and challenge of managing employee performance. There is a convincing need for organizations to develop strong and supportive management and leadership systems, particularly for frontline workers given their atypical and straining work conditions. To do so, it is prudent for managers to continuously interact and engage with these employees to offer emotional support and encouragement, address any issues of concern, and demonstrate care for these workers and their families. Embodying these managerial qualities may be central to improved employee performance during the pandemic and the mitigation of the negative employee performance outcomes that our study established.

## Figures and Tables

**Table 1:** Survey measures.

Variable	Scale Items
Perceived Risk of COVID-19	I am afraid of coronavirus
	2. Coronavirus is almost always terminal
	3. Coronavirus is an unpredictable disease
	4. I am afraid of losing my life because of coronavirus
	5. I am worried about getting infected with coronavirus
	6. My heart races when I think about getting infected with coronavirus
Psychological Distress	7. I feel stressed about the pandemic
	8. I feel that I am unable to control the important things in my life
	9. I feel as if something serious is going to happen due to the pandemic
	10. I feel upset that things related to the pandemic are out of my control
	11. I feel that the difficulties are increasing and I am unable to overcome them
Turnover Intention	12. I often think of leaving this organization
	13. I intend to look for a new job within the next year
	14. If I could choose again, I would not work for this organization
Employee Disengagement	15. I no longer feel strong and capable to do my work
	16. I can no longer continue working for a long period of time
	17. I no longer find the work that I do full of meaning
	18. I am no longer proud of the work that I do
	19. My job is challenging
Low Morale	20. I no longer feel enthusiastic about my job due to COVID-19
	21. I no longer feel energetic to do my work due to COVID-19
	22. I no longer find the work that I do full of purpose due to COVID-19
Burnout	23. I feel emotionally drained from my work
	24. I feel used up at the end of the work day
	25. I feel fatigued when I get up in the morning and have to face another day at work
	26. I feel burned out from my work
	27. I feel frustrated by my job

**Table 2:** Pearson correlations.

Correlations	Mean	SD	B	ED	LM	PRC	PD	TI
Burnout (B)	4.7765	0.35107	1					
Employee disengagement (ED)	4.7752	0.34196	0.257 **	1				
Low morale (LM)	4.8081	0.35176	0.511 **	0.348 **	1			
Perceived risk of COVID-19 (PRC)	4.7968	0.39397	0.368 **	0.342 **	0.262 **	1		
Psychological distress (PD)	4.8167	0.27799	0.532 **	0.485 **	0.452 **	0.342 **	1	
Turnover intention (TI)	4.7532	0.43338	0.431 **	0.334 **	0.380 **	0.483 **	0.380 **	1

** Correlation coefficient is significant at the 0.01 level (2-tailed).

**Table 3:** Discriminant validity using Heterotrait–Monotrait ratio (HTMT).

Construct	B	ED	LM	PRC	PD
Burnout (B)					
Employee disengagement (ED)	**0.519**				
Low morale (LM)	1.094	**0.768**			
Perceived risk of COVID-19 (PRC)	**0.736**	**0.700**	**0.614**		
Psychological distress (PD)	1.460	1.209	1.219	**0.813**	
Turnover intention (TI)	1.049	**0.831**	1.014	1.158	1.135

All HTMT values highlighted in bold have acceptable discriminant validity. Those not highlighted in bold exceed the suggested threshold.

**Table 4:** Direct effects.

Hypothesized Effect	Sample Mean	Standard Deviation	T Statistic	*p* Value	Result
PRC -> Burnout	0.239	0.052	4.3060	0.000	Positive
PRC -> Employee disengagement	0.172	0.043	3.6870	0.000	Positive
PRC -> Low morale	0.171	0.042	3.7120	0.000	Positive
PRC -> Psychological distress	0.363	0.069	5.0390	0.000	Positive
PRC -> Turnover intention	0.148	0.040	3.2960	0.001	Positive
PD -> Burnout	0.660	0.064	10.195	0.000	Positive
PD -> Employee disengagement	0.472	0.062	7.4010	0.000	Positive
PD -> Low morale	0.468	0.051	8.8530	0.000	Positive
PD -> Turnover intention	0.404	0.052	7.3110	0.000	Positive

**Table 5:** Indirect mediation effects.

Hypothesized Effect	Sample Mean	Standard Deviation	T Statistic	*p* Value	Result
PRC -> PD -> Burnout	0.239	0.052	4.3060	0.000	Positive
PRC -> PD -> Employee disengagement	0.172	0.043	3.6870	0.000	Positive
PRC -> PD-> Low morale	0.171	0.042	3.7120	0.000	Positive
PRC -> PD -> Turnover intention	0.148	0.040	3.2960	0.001	Positive

## Data Availability

The data presented in this study is available upon request from the corresponding author.
